# Comparison of Outcomes of Active Surveillance in Intermediate-Risk Versus Low-Risk Localised Prostate Cancer Patients: A Systematic Review and Meta-Analysis

**DOI:** 10.3390/jcm12072732

**Published:** 2023-04-06

**Authors:** Subhabrata Mukherjee, Dimitrios Papadopoulos, Joseph M. Norris, Mudassir Wani, Sanjeev Madaan

**Affiliations:** 1 Department of Urology, Charing Cross Hospital, Imperial College Healthcare NHS Trust, Fulham Palace Rd, London W6 8RF, UK; 2 Department of Urology, West Middlesex University Hospital, Chelsea and Westminster Hospital NHS Foundation, Twickenham Rd, Isleworth TW7 6AF, UK; 3 Department of Urology, Swansea Bay University Health Board, Swansea SA6 6NL, UK; 4 Department of Urology, Dartford and Gravesham NHS Trust, Dartford DA2 8DA, UK

**Keywords:** prostate cancer, active surveillance, active monitoring, low-risk prostate cancer, intermediate-risk prostate cancer

## Abstract

Currently, there is no clear consensus regarding the role of active surveillance (AS) in the management of intermediate-risk prostate cancer (IRPC) patients. We aim to analyse data from the available literature on the outcomes of AS in the management of IRPC patients and compare them with low-risk prostate cancer (LRPC) patients. A comprehensive literature search was performed, and relevant data were extracted. Our primary outcome was treatment-free survival, and secondary outcomes were metastasis-free survival, cancer-specific survival, and overall survival. The DerSimonian–Laird random-effects method was used for the meta-analysis. Out of 712 studies identified following an initial search, 25 studies were included in the systematic review. We found that both IRPC and LRPC patients had nearly similar 5, 10, and 15 year treatment-free survival rate, 5 and 10 year metastasis-free survival rate, and 5 year overall survival rate. However, cancer-specific survival rates at 5, 10, and 15 years were significantly lower in IRPC compared to LRPC group. Furthermore, IRPC patients had significantly inferior long-term overall survival rate (10 and 15 year) and metastasis-free survival rate (15 year) compared to LRPC patients. Both the clinicians and the patients can consider this information during the informed decision-making process before choosing AS.

## 1. Introduction

Worldwide, prostate cancer (PC) at 14.1% is the second most frequently occurring cancer in men and is closely behind the most commonly occurring lung cancer (14.3%) [1]. The incidence of PC diagnosis has markedly increased over the last 30 years with the widespread use of screening programmes featuring prostate-specific antigen (PSA) as a tumour marker [2]. As expected, a large proportion of new diagnoses consist of low-risk and intermediate-risk disease with a relatively low potential for rapid progression and cancer-specific death [3,4]. As definitive treatments such as radical prostatectomy or radiotherapy are not free of complications and significantly impact patients’ quality of life related to sexual, urinary, and bowel function [5], therein lies the dilemma of whether or not to actively treat those PCs which are supposed to be slow-growing and less likely to manifest in the lifetime of the patients. This is why many national institutions have decided against routine PSA screening aiming to reduce indolent PC diagnosis and the harm from unnecessary treatment [6,7]. Towards the same direction, a monitoring approach for early PC has been incorporated in standard practice and has been used for more than 20 years [8].

Active surveillance (AS) or deferred treatment involves close disease monitoring with regular PSA testing, digital rectal examination (DRE), magnetic resonance imaging (MRI), and prostate biopsies at various times during the follow-up until a predefined stage, suggesting that early evidence of disease progression is reached or patients become unfit for any major interventions due to advanced age or comorbidities [9]. The AS strategy is aimed at avoiding overtreatment, reducing the associated side-effects of treatment, and maintaining the quality of life of patients, with an intention to offer active intervention without compromising the window of opportunity for curative treatment [10,11].

AS has been a common strategy and is well recommended in low-risk prostate cancer (LRPC) patients with equivalent oncological outcomes [8,12,13]. However, when it comes to intermediate-risk prostate cancer (IRPC) patients, there is a general hesitancy due to the ambiguous nature of the available literature [14,15]. Available guidelines are also not uniform in this regard. For example, National Institute for Health and Care Excellence (NICE) guidelines recommend AS as an option for IRPC patients without defining clear patient selection criteria [16]. National Comprehensive Cancer Network (NCCN) guidelines advise that AS could be used in well-informed favourable IRPC patients with caution and close monitoring [17]. With respect to unfavourable IRPC, NCCN guidelines recommend against AS due to its more aggressive behaviour. European Association of Urology (EAU) guidelines, in the same context, weakly recommend AS in highly selected IRPC patients with low-volume International Society of Urological Pathology (ISUP) Grade Group (GG) 2 disease or another single component of intermediate-risk disease with low disease extent [18]. Therefore, there is no clear consensus regarding the role of AS in the management of IRPC patients.

In this systematic review and meta-analysis, we aimed to extract and analyse data from the available literature on the outcomes of AS in the management of IRPC patients and compare them with LRPC patients to help the clinicians and the patients in informed decision making before choosing AS.

## 2. Materials and Methods

### 2.1. Protocol and Registration

The protocol for this systematic review was written according to the guidance set out in the PRISMA (Preferred Reporting Items for Systematic Reviews and Meta-Analyses) Protocols 2015 checklist [19] and was prospectively registered in the PROSPERO International Registry (CRD42022354861).

### 2.2. Review Question

Our review question sought to determine how the outcomes of IRPC patients managed with AS differ from LRPC patients managed similarly. We framed our review question according to the PICO (population, intervention, control, and outcomes) criteria [20] as follows: population—localised IRPC and LRPC patients; intervention—AS; comparison—IRPC versus LRPC patients; outcome (primary)—treatment-free survival, and outcomes (secondary)—metastasis-free survival, cancer-specific survival, and overall survival.

### 2.3. Inclusion and Exclusion Criteria

Original articles discussing the outcomes of IRPC patients with or without LRPC patients managed by AS were included in the review, whereas original articles discussing the AS outcomes in only LRPC patients or without risk group subclassification were excluded. Furthermore, review articles, conference abstracts, case reports, expert opinions, and non-English language articles were excluded.

### 2.4. Search Methodology

A comprehensive literature search was performed on the PubMed, Embase, and Cochrane databases to find all pertinent studies published from July 1992 (date of first reported series of conservative management of localised prostate cancer) [21] to October 2022. To reduce the number of unrelated studies, controlled vocabulary was selected in the search engines. The search strategy contained 14 components linked by the AND/OR operator terms: (prostat* cancer OR PCa OR prostat* adenocarcinoma OR prostat* malignancy OR prostat* tumo*) AND (active surveillance OR AS OR active monitoring) AND (low risk) AND (intermediate risk) AND (strategy OR regime OR monitoring OR pathway).

### 2.5. Study Selection

First, all the identified articles were uploaded to Rayyan (a web and mobile application for systematic reviews) to remove duplicates, facilitate initial screening, and improve collaboration. Thereafter, using the title and abstract, the available studies were screened. Subsequently, the full-text articles were scrutinised against the inclusion and exclusion criteria before selecting the final articles for the systematic review. Moreover, reference lists for each included article were screened manually to reduce the risk of missing relevant studies. The entire process was carried out independently by two separate reviewers (S.M. (Subhabrata Mukherjee). and D.P.). Any disagreements were resolved after discussion with the remaining authors (J.M.N., M.W. and S.M. (Sanjeev Madaan)), and a consensus was reached.

### 2.6. Data Extraction

All the included articles were meticulously read by all authors. Relevant data points were extracted and organised onto a spreadsheet by two separate reviewers (S.M. (Subhabrata Mukherjee) and D.P.) independently.

### 2.7. Risk-of-Bias Assessment

Risk-of-bias assessment was conducted using the Newcastle–Ottawa scale [22,23]. Studies were assessed on grounds of patient selection, comparability, and outcome. The Newcastle–Ottawa scale allowed for a maximum of nine stars: four for selection, two for comparability, and three for outcome. A total score of ≤5 was considered low quality, 6–7 was considered intermediate quality, and 8–9 was considered high quality.

### 2.8. Statistical Analysis

Meta-analysis methods were used to pool together the within-study differences in outcome between IRPC and LRPC patients. The main outcomes were measured at different timepoints. Separate analyses were performed at each follow-up timepoint. All outcomes were binary in nature, and the differences between groups were expressed as a relative risk.

The analysis was performed using Stata (version 15.1). The DerSimonian–Laird random-effects method was used for the analysis, regardless of the degree of heterogeneity between the study results. Heterogeneity between studies was assessed on the basis of the significance of the between-study heterogeneity, as well as the size of the I^2^ value. Substantial heterogeneity was assumed if the I^2^ value was above 50%.

## 3. Results

### 3.1. Study Selection Results

**Figure 1:**
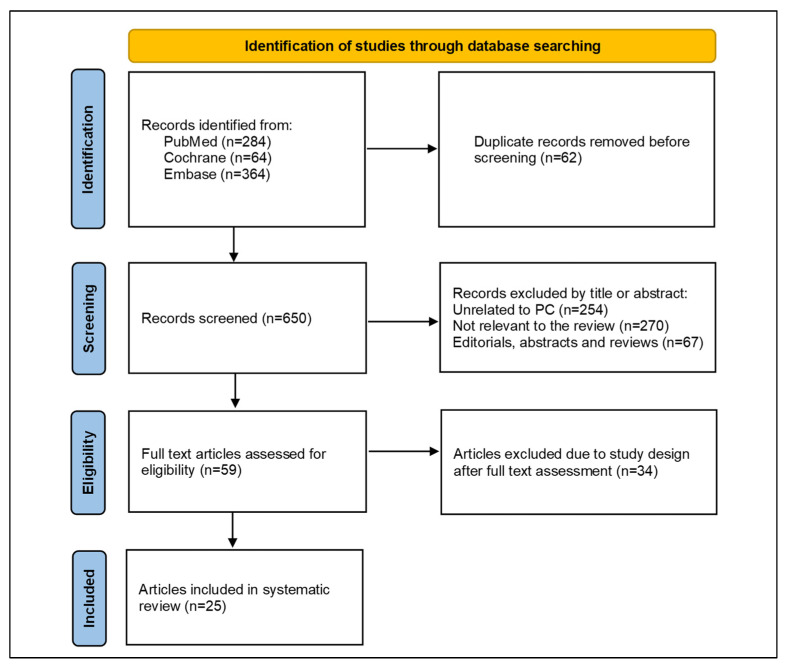
The Preferred Reporting Items for Systematic Reviews and Meta-Analyses (PRISMA) flow diagram of study selection.

Out of 712 studies identified following the initial search, 62 were removed to avoid duplication; therefore, 650 studies were screened (Figure 1). Of these, scrutinising against the selection criteria, 591 studies were excluded after reading the title or abstract, and 34 studies were excluded after full-text review. Ultimately, 25 studies were included in the systematic review [11,14,15,24,25,26,27,28,29,30,31,32,33,34,35,36,37,38,39,40,41,42,43,44,45].

### 3.2. Bias Assessment Results

The majority of included trials (20/25; 80%) scored highly on the Newcastle–Ottawa scale, indicating an acceptably low risk of bias (Table 1). A small proportion of studies (5/25; 20%) scored intermediate on the scale. All studies scored highly on patient selection; however, quality scores varied in comparability and outcome ascertainment domains, due to an inadequate description of the exclusion criteria, record linkage, or investigator blinding.

### 3.3. Study Characteristics

The systematic review incorporating 25 studies discussed the outcomes of 29,959 IRPC and 49,833 LRPC patients. As expected, there were some variations in patient selection criteria. The majority of the studies (15 out of 25) used National Comprehensive Cancer Network (NCCN) criteria to define the risk groups. However, one study used D’Amico criteria, two used the Cancer of the Prostate Risk Assessment (CAPRA) score (LRPC—CAPRA score 0–2, IRPC—CAPRA score 3–5), and three used the histology Gleason grade (LRPC—GG 1, IRPC—GG 2 or 3) for risk group definition. The remaining four studies made their own modifications to define IRPC and LRPC. Details are included in Supplementary Table S1. There were also some differences in the active surveillance protocol and trigger point for offering deferred treatment between the studies. Only six studies mentioned MRI in the AS protocol. A summary of the methodology, baseline characteristics of the study population, and follow-up periods with survival data of the included studies are shown in Supplementary Tables S1–S3, respectively.

### 3.4. Meta-Analysis Results

All analyses considered the difference in outcomes between the two risk groups. For the purposes of analysis, for each study, all intermediate-risk groups were combined, and similarly all low-risk groups (including very-low-risk groups) were combined.

A summary of the meta-analysis results is presented in Table 2. The figures describe the studies with valid data included in each analysis. Subsequently, details of the heterogeneity are reported, in terms of both the significance and the I^2^ values. The final columns give the differences in outcome between the two risk groups. The pooled difference in outcome between groups is expressed as a relative risk. This gives the occurrence of the outcome in the intermediate group relative to the risk of the outcome in the low-risk group. The pooled figure is presented along with a corresponding confidence interval. The final column shows the *p*-values indicating the significance of the differences between risk groups.

The degree of heterogeneity between studies varied for the different outcomes. However, there was generally a high degree of heterogeneity between studies, with significant heterogeneity tests and high I-squared values.

#### 3.4.1. Deferred Treatment

**Figure 2:**
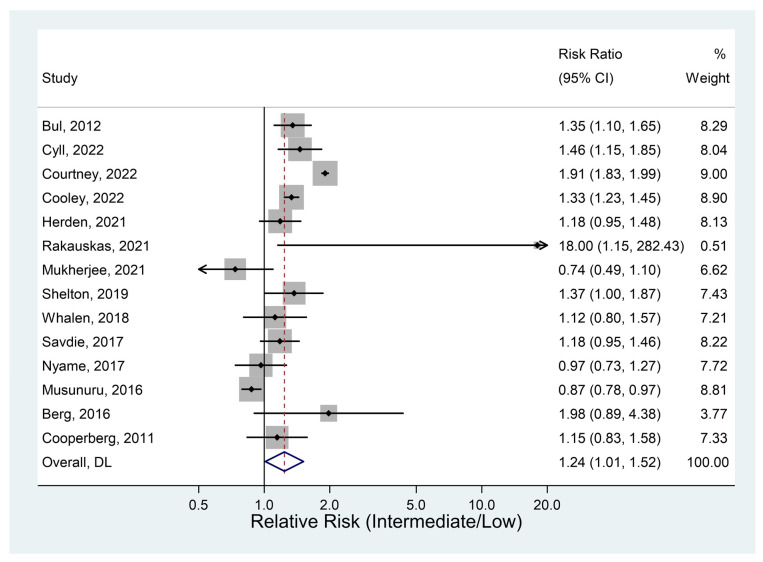
Definitive treatment (all studies) [11,25,26,28,29,30,31,35,38,39,40,41,42,45].

Fourteen studies provided data on the deferred treatment rate. The analysis suggested slight evidence that definitive treatment varied between groups, although the result was only of borderline statistical significance (Figure 2). There was a trend towards definitive treatment being more common in the intermediate-risk group. With all studies included, the chance of definitive treatment was 24% higher for the intermediate group than the low-risk group. As there was a very large difference for one study [30], the analysis was repeated with this study omitted. The results were not greatly changed by the omission of this study.

#### 3.4.2. Treatment-Free Survival

**Figure 3:**
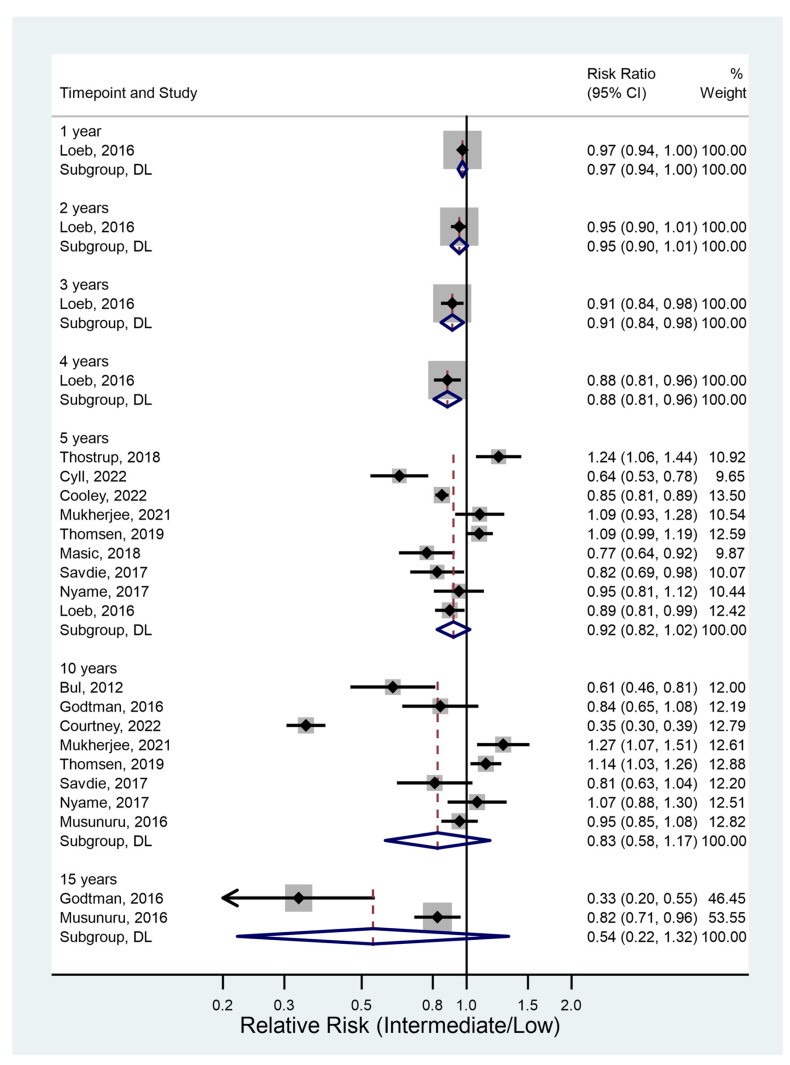
Treatment-free survival [11,14,15,25,26,28,31,36,37,39,40,41,44].

Thirteen studies had relevant data. There was some evidence that treatment-free survival was significantly different between groups for the 1, 2, 3, and 4 year data (Figure 3). The results reached statistical significance on years 3 and 4, and they were of borderline significance for years 1 and 2. Treatment-free survival was lower in the intermediate-risk group, with the chance of survival at 4 years only 88% as high as in the low-risk group.

At 5 years (RR 0.92, 95% CI 0.82–1.02, *p* = 0.12), 10 years (RR 0.83, 95% CI 0.55–1.23, *p* = 0.35), and 15 years (RR 0.54, 95% CI 0.21–1.39, *p* = 0.20) there was no real evidence that treatment-free survival varied significantly between the two groups.

#### 3.4.3. Metastasis-Free Survival

**Figure 4:**
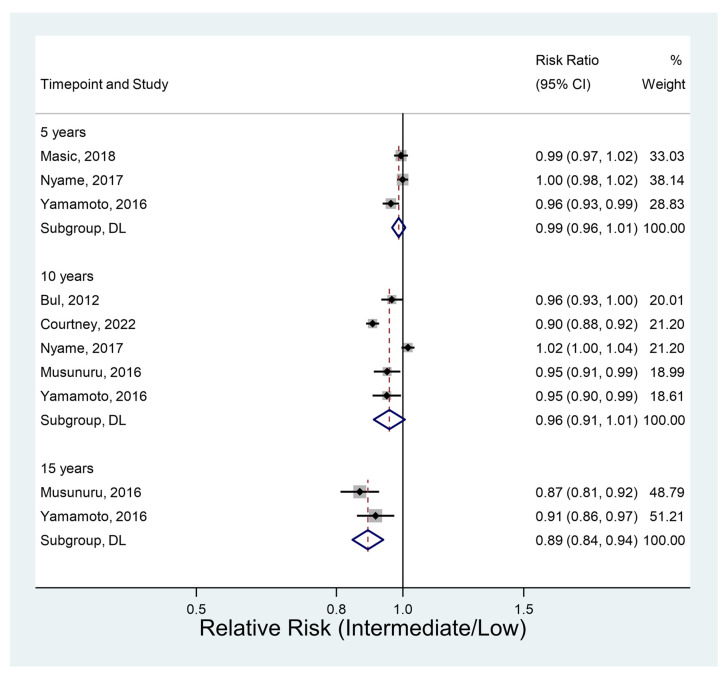
Metastasis-free survival [11,26,37,40,41,43].

Six studies had data on metastasis-free survival. It did not vary significantly between groups at either 5 or 10 years (Figure 4). However, the two studies providing data at 15 years (RR 0.89, 95% CI 0.84–0.94, *p* < 0.001) suggested a significantly low survival rate in the intermediate-risk group. Metastasis-free survival at this point was over 10% lower than in the low-risk group.

#### 3.4.4. Cancer-Specific Survival

**Figure 5:**
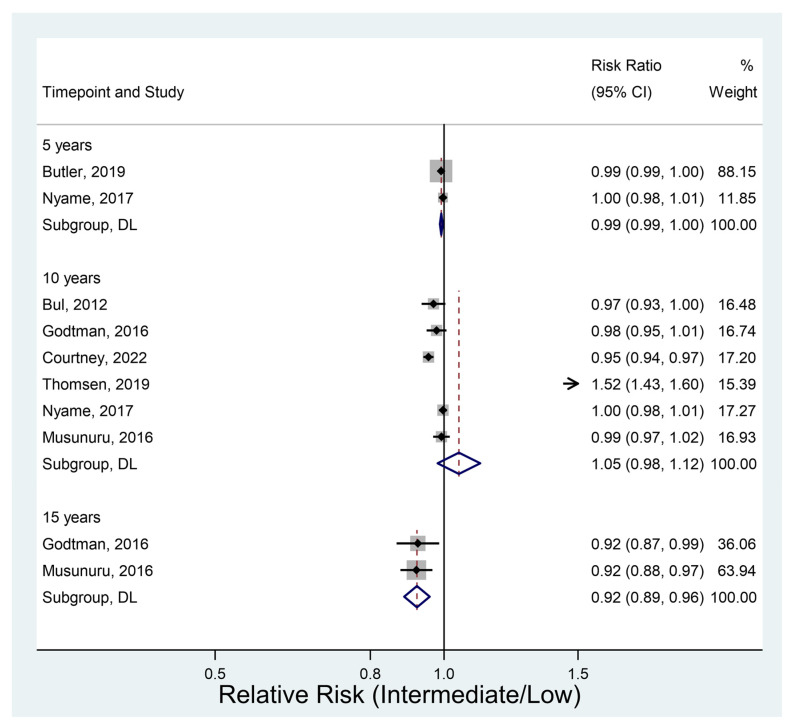
Cancer-specifical survival [11,14,26,34,36,40,41].

Six studies had relevant data. Cancer-specific survival was significantly lower in the intermediate-risk group than in the low-risk group at all three timepoints (Figure 5). Survival was 8% lower in the intermediate-risk group at 15 years (RR 0.92, 95% CI 0.89–0.96, *p* < 0.001).

#### 3.4.5. Overall Survival

**Figure 6:**
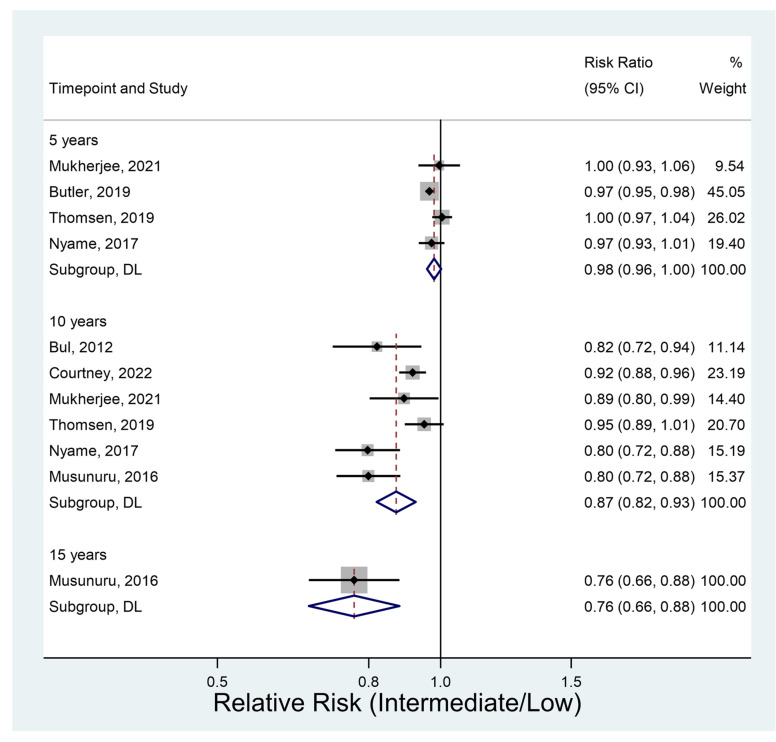
Overall survival [11,26,31,34,36,40,41].

Seven studies provided data on overall survival. It did not vary significantly between groups at 5 years (Figure 6). However, a significant difference at both 10 and 15 years was observed with significantly lower overall survival in the intermediate-risk group. Survival at 10 years (RR 0.87, 95% CI 0.82–0.93, *p* < 0.001) was 13% lower in the intermediate-risk group than in the low-risk group.

## 4. Discussion

### 4.1. AS Outcomes—IRPC vs. LRPC

Our systematic review and meta-analysis examining the outcomes of AS in IRPC patients found that, although there was a trend towards definitive treatment being more common in the intermediate-risk group, there was no significant difference in the treatment-free survival rate at 5 years (RR 0.92, 95% CI 0.82–1.02, *p* = 0.12), 10 years (RR 0.83, 95% CI 0.55–1.23, *p* = 0.35), and 15 years (RR 0.54, 95% CI 0.21–1.39, *p* = 0.20) between IRPC and LRPC patients. Furthermore, there was no significant difference in the 5 and 10 year metastasis-free survival rate and 5 year overall survival rate between the groups. However, IRPC patients had significantly lower 15 year metastasis-free survival, 10 and 15 year overall survival, and 5, 10, and 15 year cancer-specific survival compared to the LRPC group. It is of note that the conclusion on the difference in 15 year overall survival was made from only one study [41], which included patients from 1995–2013 and, therefore, likely does not represent the contemporary era of subsequent therapies that may significantly prolong survival. Similarly, conclusions on the remaining 15 year survival rates were drawn from two studies only as long-term data were limited.

### 4.2. AS Outcomes—IRPC without GG 3 Disease vs. LRPC

Eleven studies did not include GG 3 patients in their study population. Of these, Courtney et al. [26] and Mukherjee et al. [31] revealed separate data on metastasis-free survival, cancer-specific survival, and overall survival between the two groups.

Courtney et al. [26] observed AS outcomes of 773 FIRPC and 8726 LRPC patients over a median period of 7.6 years. They noticed significantly lower 10 year metastasis-free survival (90.4% vs. 98.5%, *p* = 0.001) and 10 year cancer-specific survival (96.3% vs. 98.9%, *p* = 0.001) in FIRPC compared to LRPC. However, there was no difference in 10 year overall survival between the groups (73.8% vs. 76.8%, *p* = 0.13).

Mukherjee et al. [31], managed 96 IRPC patients without GG 3 disease and 276 LRPC patients by AS for a median period of 4.5 years. Biochemical recurrence was noted in one IRPC patient and eight LRPC patients. Only one patient from the LRPC group developed metastasis. There was no difference in the overall survival (5 years 93% vs. 93%; 10 years 83% vs. 90%; HR 0.79, 95% CI 0.25–2.47) between IRPC and LRPC groups.

### 4.3. AS Outcomes—GG 2 Disease vs. GG 1 Disease

Masic et al. [37] retrospectively reviewed the outcomes of 1119 GG 1 and 124 GG 2 PC patients who were managed by AS between 1990 and 2016. GG 2 patients had significantly lower 5 year treatment-free survival (49% vs. 64%, *p* < 0.01) than GG 1 patients. GG 2 patients also had significantly lower unadjusted 2 year biochemical recurrence-free survival (69% vs. 93%, *p* = 0.01) and a higher recurrence risk (HR 3.67, 95% CI 1.30–10.36). However, there was no difference in the risk of metastasis between the groups. There was no prostate cancer-specific mortality. In sensitivity analyses, multiple GG 2 cores were associated with a higher risk of deferred treatment.

### 4.4. AS Outcomes—In Only IRPC Groups

Carlsson et al. [32], Richard et al. [33], and Sayyid et al. [27] discussed the outcomes of AS in IRPC patients only. Carlsson et al. [32] managed 219 GG 2 PC patients by AS (median follow-up 3.1 years); 64 of them (29%) needed deferred treatment. Treatment-free survival at 5 and 10 years was 61% (95% CI 52–70) and 49% (95% CI 37–60), respectively. Only three patients developed biochemical recurrence, but none of them developed metastasis or died of PC. Overall survival at 5 years was 97% and at 10 years was 77%.

Richard et al. [33] reported outcomes of 374 GG 2 and GG 3 PC patients who were managed by AS between 2002 and 2011. Compared to Carlsson et al., they noted a higher deferred treatment rate of 71% (266 out of 374 patients) after 8 years follow-up with a 5 year treatment-free survival of 35%. However, 5 year and 8 year cancer-specific survival was 98% and 94%, respectively. Overall survival was 94% at 5 years and 82% at 8 years.

Lastly, Sayyid et al. [27] searched the Surveillance, Epidemiology, and End Results (SEER) Prostate with Watchful Waiting database and identified 20,334 FIRPC patients who were managed by AS between 2010 and 2015. Their observed deferred treatment rate was as high as 88% (17,895 out of 20,334 men). They found that patients with higher clinical stage, cancer volume, or socioeconomic status were significantly more likely to have deferred treatment.

### 4.5. AS Outcomes—Based on the Age of Diagnosis

Ventimiglia et al. [24]. Who retrospectively reviewed long-term outcomes of AS in 16,177 men from the Prostate Cancer data Base Sweden (PCBaSe), found a growing benefit of AS with increasing age at diagnosis and decreasing risk group. However, there was no great difference in treatment-free survival between IRPC and LRPC groups irrespective of the age of diagnosis. When diagnosed at age 55 years, the mean proportion of remaining life-years without definitive treatment in the IRPC group was 7 of 25 years (29%) vs. 9 of 25 years (36%) in the LRPC group. When diagnosed at 70 years, the figures changed to 8 of 13 years (60%) in the IRPC population vs. 9 of 13 years (66%) in the LRPC population. Moreover, age of diagnosis did not have a large impact on the cancer-specific mortality between the risk groups. The risk of death from prostate cancer before age 85 years when diagnosed at age 55 years was 15% in the IRPC group vs. 13% in the LRPC group. When diagnosed at 70 years, the figures changed to 7% in the IRPC group vs. 6% in the LRPC group. IRPC patients who were diagnosed before age 60 years had higher cancer-specific mortality risk (12%−15%) and lower remaining life-years without definitive treatment (29%−33%) compared to LRPC men diagnosed above the age of 65 years (cancer-specific mortality risk: 3−5%; remaining life-years without definitive treatment: 62−77%). The authors concluded that AS may be a safe strategy for disease management among men with low- to intermediate-risk PC who were older than 65 years at diagnosis.

### 4.6. AS Outcomes—Other Reviews

Enikeev et al. [46], who analysed 17 articles in their systematic review and meta-analysis, noted oncological outcomes grossly similar to ours except that they did not find any difference in 5 year cancer-specific survival but observed a significantly lower 10 year metastasis-free survival (OR 0.46, 95% CI 0.28–0.77, *p* = 0.003) in the IRPC group compared to the LRPC group.

Likewise, another systematic review and meta-analysis of 25 studies by Baboudjian et al. [47] noted no significant difference in the risk of deferred treatment (RR 1.16, 95% CI 0.99–1.36, *p* = 0.07), but significantly higher risks of metastasis (RR 5.79, 95% CI 4.61–7.29, *p* < 0.001), cancer-specific mortality (RR 3.93, 95% CI 2.93–5.27, *p* < 0.001), and overall mortality (RR 1.44, 95% CI 1.11–1.86, *p* = 0.005) in IRPC patients compared to LRPC patients.

### 4.7. What This Study Adds

Results of our study contribute to the growing body of literature discussing the oncological outcomes of AS in IRPC patients and compared them with the LRPC group. To our knowledge, this is the most up-to-date systematic review and meta-analysis on the topic. Findings of our study should help in informed decision making before choosing AS in IRPC patients.

### 4.8. Future Direction

Even in the heterogenous group of IRPC patients including high-volume GG 2 disease and GG 3 disease without routine pre-biopsy MRI scan, there was no significant difference in treatment-free survival up to 15 years, metastasis-free survival up to 10 years, and overall survival up to 5 years compared to LRPC patients. Therefore, we can probably expect that the present difference between the two groups would be minimised in the modern AS series where patients have a routine upfront MRI scan followed by systematic and targeted biopsies, and where only low-volume GG 2 IRPC patients are included for AS [48,49]. In addition, use of novel biomarkers and molecular genetics [50,51] in assessing disease aggressiveness and selecting appropriate IRPC candidates for AS would further contribute to better oncological outcomes.

### 4.9. Limitations

Firstly, there was substantial heterogeneity in the definition of IRPC, follow-up protocol, use of MRI, trigger point for intervention, and follow-up period between the studies, which may have influenced the meta-analysis result. Secondly, although the volume of GG 2 disease appears to be an important predictor of adverse AS outcome, it was not possible to subclassify IRPC patients on the basis of GG 2 disease volume, as separate data were not available. Thirdly, it was also not possible to assess the outcome of IRPC patients who had a pre-biopsy MRI scan followed by systematic and targeted biopsy due to the paucity of available separate data. Lastly, only four of the included 25 studies had 15 year survival data; considering the slow-growing nature of the disease, we consider the lack of long-term data as one of the limitations.

### 4.10. Strengths

Overall, our review methodology seemed to be appropriate and robust. The study protocol was prospectively registered in the PROSPERO International Registry (CRD42022354861) with predefined review question according to the PICO (population, intervention, control, and outcomes) criteria and objective inclusion and exclusion criteria for study selection. A systematic and comprehensive literature search was performed in multiple databases with a predefined search strategy. Additional articles were identified through manual screening of the relevant bibliography, and the search strategy was reported as per the PRISMA statement. Study selection and data extraction were performed independently by two reviewers, and any disagreements were resolved by consensus. The review analysed 25 of the most up-to-date articles including 29,959 IRPC and 49,833 LRPC patients. The study methodology, patient characteristics, and survival outcomes of each included article were illustrated with adequate details in table format. The DerSimonian–Laird random-effects method was used by an independent statistician for the meta-analysis, regardless of the degree of heterogeneity between the study results. Lastly, the oncological outcomes of AS were presented in various possible ways for better explanation and understanding.

## 5. Conclusions

In conclusion, our study suggested that, compared to the LRPC group, IRPC patients managed by AS have a nearly similar long-term treatment-free survival rate, as well as short- to intermediate-term metastasis-free survival rate and overall survival rate. However, IRPC patients have a significantly lower cancer-specific survival rate than LRPC patients, even in the short term. Both clinicians and patients can consider this information during the informed decision-making process before opting for AS. It is expected that routine pre-biopsy MRI scan, inclusion of only low-volume GG 2 disease or favourable IRPC, and use of novel biomarkers in patient selection would contribute to better oncological outcomes and probably reduce the present difference between the groups.

## Figures and Tables

**Table 1:** Newcastle–Ottawa scale quality scores for included studies.

Study	Score	Cohort Selection	Comparability	Outcome Ascertainment
Ventimiglia, 2022 [24]	8	4	1	3
Cyll, 2022 [25]	8	4	2	2
Courtney, 2022 [26]	9	4	2	3
Sayyid, 2022 [27]	9	4	2	3
Cooley, 2021 [28]	8	4	1	3
Herden, 2021 [29]	8	4	2	2
Rakauskas, 2021 [30]	9	4	2	3
Mukherjee, 2021 [31]	9	4	2	3
Carlsson, 2020 [32]	8	4	2	2
Richard, 2020 [33]	8	4	2	2
Butler, 2019 [34]	9	4	2	3
Shelton, 2019 [35]	8	4	2	2
Thomsen, 2019 [36]	7	4	1	2
Masic, 2018 [37]	8	4	2	2
Whalen, 2018 [38]	6	4	1	1
Thostrup, 2018 [15]	7	4	1	2
Savdie, 2017 [39]	8	4	2	2
Nyame, 2017 [40]	7	3	1	3
Musunuru, 2016 [41]	8	4	2	2
Godtman, 2016 [14]	9	4	2	3
Berg, 2016 [42]	8	4	2	2
Yamamoto, 2016 [43]	7	4	1	2
Loeb, 2016 [44]	9	4	2	3
Bul, 2012 [11]	9	4	2	3
Cooperberg, 2011 [45]	8	4	2	2

**Table 2:** Summary of meta-analysis results.

Outcome	Outcome	Number	Heterogeneity		Group Difference	
	Time	Studies	*p*-Value	I^2^	RR (95% CI) ^(*)^	*p*-Value
Deferred trt	-	14	<0.001	96%	1.24 (1.00, 1.53)	0.05
Deferred trt ^(+)^	-	13	<0.001	96%	1.22 (0.99, 1.51)	0.06
Treatment-free	1 year	1	-	-	0.97 (0.95, 1.00)	0.07
survival	2 years	1	-	-	0.95 (0.90, 1.01)	0.08
	3 years	1	-	-	0.91 (0.84, 0.98)	0.01
	4 years	1	-	-	0.88 (0.81, 0.96)	0.005
	5 years	9	<0.001	87%	0.92 (0.82, 1.02)	0.12
	10 years	8	<0.001	98%	0.83 (0.55, 1.23)	0.35
	15 years	2	<0.001	92%	0.54 (0.21, 1.39)	0.20
Metastasis-free	5 years	3	0.02	73%	0.99 (0.96, 1.01)	0.27
survival	10 years	5	<0.001	95%	0.96 (0.90, 1.02)	0.16
	15 years	2	0.25	25%	0.89 (0.84, 0.94)	<0.001
Cancer-specific	5 years	1	-	-	0.99 (0.99, 0.99)	<0.001
survival	10 years	4	0.04	64%	0.97 (0.95, 0.99)	0.008
	15 years	2	0.91	0%	0.92 (0.89, 0.96)	<0.001
Overall survival	5 years	4	<0.001	32%	0.98 (0.94, 1.02)	0.21
	10 years	6	<0.001	63%	0.87 (0.82, 0.93)	<0.001
	15 years	1	-	-	0.76 (0.66, 0.88)	<0.001

^(*)^ Relative risks expressed as outcome for Intermediate group divided by outcome for Low group. ^(+)^ Analysis omitting data from one study (Rakauskas).

## Data Availability

The data presented in this study are available on request from the corresponding author. The data are not publicly available due to privacy.

## Supplementary Materials

The following supporting information can be downloaded at https://www.mdpi.com/article/10.3390/jcm12072732/s1: Table S1. Summary of methodology of the included studies; Table S2. Baseline characteristics of the different study populations; Table S3. Follow-up period and survival data of the different study populations.
