# Supplementary material for: Comparison of Outcomes of Active Surveillance in Intermediate-Risk Versus Low-Risk Localised Prostate Cancer Patients: A Systematic Review and Meta-Analysis
Source: J Clin Med. 2023 Apr 6;12(7):2732. doi: 10.3390/jcm12072732 (PMC10094761; doi:10.3390/jcm12072732)
Supplement: Supplementary file 1 [file jcm-12-02732-s001.zip › jcm-2219876-supplementary.pdf]

Table S1. Summary of methodology of the included studies.

Study name	Study period	Study design	Risk stratification	MRI scan	Follow up confirmatory biopsy	Trigger for intervention
Ventimiglia (2022 Sep) [24]	1992-2014	Retrospective	VLRPC: T1c, positive cores ≤ 33%, cancer length ≤8mm, GS 6, PSA <10, prostate volume <90cc, >5 core biopsies performed, PSAD < 0.15.<br>LRPC: GS 6; PSA <10; T1/T2 with at least one of the following: prostate volume ≥90cc, PSAD ≥0.15, ≤5 core biopsies performed, positive cores > 33%, cancer length > 8 mm; or GGG 1, T1 or T2 with PSA 10-14.<br>IRPC: GS 7 (3+4), PSA <10, T1 or T2	NA	NA	NA
Cyll (2022 Apr) [25]	2009-2016	Prospective	LRPC: CAPRA score 0-2 and PSA <10 and GGG 1.<br>IRPC: CAPRA score 3-5 and/or PSA 10-20 and/or GGG 2	Most patients had MRI before or within one year of diagnosis.<br>LRPC: MRI after 12, 48 and 60 months.<br>IRPC: MRI after 12, 24 and 48, and every second year after that.	LRPC: At 1 year and every 60 months thereafter.<br>IRPC: At 1 and 2 years, and every 60 months thereafter.	Histological reclassification (GGG ≥ 3, perineural invasion or increase in number of positive biopsies), radiological reclassification (EPE or SVI, increase in tumour diameter or the number of PI-RADS score > 3 lesions), PSA re-classification (PSA >20 or PSADT <1 year). Clinical reclassification (cT ≥3), patient preference
Courtney (2022 Feb) [26]	2001-2015	Retrospective	As per NCCN criteria	NA	At least once	NA

Sayyid (2022 Jan) [27]	2010-2015	Retrospective	As per NCCN criteria	NA	NA	NA
Cooley (2021 Nov) [28]	1991-2018	Prospective	As per NCCN criteria	NA	NA	Grade re-classification, PSA progression, tumour volume progression, anxiety
Herden (2021 Jul) [29]	2008-2013	Prospective	As per NCCN criteria	NA	After 1 year, then every 3 years	Histologic upgrade, PSADT <3 years, clinical T stage upgrade, patient desire
Rakauskas (2021 Jul) [30]	2013-2018	Prospective	LRPC (strict criteria): PSA <10, GS 6, number of positive biopsies ≤3, maximum cancer burden ≤3 mm and <50% invasion, mpMRI PI-RADS score 1-3.<br>FLRPC (expanded criteria): PSA <15, GS ≤3+4, number of positive biopsies ≤5, maximum cancer burden ≤8, mpMRI PI-RADS score 4-5.	At enrolment and subsequently once a year for the first two years, every 2 years thereafter	Once a year for the first two years	Progression beyond the set criteria, patient desire
Mukherjee (2021 Jan) [31]	2002-2019	Prospective	As per D'Amico criteria	At enrolment and subsequently every 2 years (since 2014). Also, if rising PSA or change in DRE findings.	At 1 year	Clinical, histological or radiological progression and patient desire

Carlsson (2020 Jun) [32]	2000-2017	Retrospective	IRPC: GG 2	At enrolment and every 18 months (contemporary strategy)	Every 2-3 years	Evidence of disease progression and patient desire
Richard (2020 Jun) [33]	2002-2011	Retrospective	IRPC: GG 2 and 3	No	NA	Evidence of disease progression and patient desire
Butler (2019 Sep) [34]	2010-2015	Retrospective	As per NCCN criteria	NA	NA	NA
Shelton (2019 Aug) [35]	2013-2017	Retrospective	As per NCCN criteria	NA	NA	Increase in GS or disease volume (≥3 cores), rising PSA, concerning findings on genetic testing, patient desire
Thomsen (2019 Mar) [36]	2002-2012	Retrospective	As per NCCN criteria	NA	At 2 years for IRPC	NA
Masic (2018 Oct) [37]	1990-2016	Retrospective	LRPC: GG 1<br>IRPC: GG 2	Used in selected patients later in the study, but not a formal part of the protocol	At 1 year, then every 1-2 years	Mainly histological upgrade
Whalen (2018 Apr) [38]	1990-2012	Retrospective	As per NCCN criteria	NA	NA	NA
Thostrup (2018 Feb) [15]	2002-2017	Prospective	As per NCCN criteria	NA	At 1 year	Clinical, pathological or PSA progression
Savdie (2017 Oct) [39]	1993-2014	Retrospective	As per NCCN criteria	Incorporated since 2010	At 18 months, then every 1-2 years	Persistent PSA rise; pathological, clinical or MRI progression; patient desire

Nyame (2017 Sep) [40]	2002-2015	Retrospective	As per NCCN criteria	NA	At 1 year, thereafter every 1-3 years	Disease reclassification (increase in grade or volume), patient desire
Musunuru (2016 Dec) [41]	1995-2013	Prospective	LRPC: cT1-T2b, GS 6, PSA ≤10<br>IRPC: cT2c, GS ≤3+4, PSA ≤15, Age >70	NA	At 6-12 months, thereafter every 3-4 years	Histologic or clinical progression (palpable nodule), PSA rise
Godtman (2016 Nov) [14]	1995-2014	Prospective	As per NCCN criteria	NA	Every 2-3 years	Disease progression (PSA, histologic grade, and/or stage) or patient initiative
Berg (2016 May) [42]	2002-2015	Prospective	As per NCCN criteria	NA	No	Radiological or histologic progression, patient preference
Yamamoto (2016 May) [43]	1995-2013	Prospective	As per NCCN criteria	NA	At 1 year, then every 3-4 years	PSA doubling time <3 years, histologic upgrade, clinical progression
Loeb (2015 Feb) [44]	2003-2007	Prospective	As per NCCN criteria	NA	NA	Histologic upgrade, short PSA doubling time, patient desire
Bul (2012 Dec) [11]	1993-2007	Prospective	LRPC: T1c/T2, PSA ≤ 10, PSAD <0.2, GS ≤6, maximum 2 positive cores.<br>IRPC: PSA 10-20, GS 7, 3 positive cores	NA	NA	Disease progression or patient desire
Cooperberg (2011 Jan) [45]	1995-2010	Prospective	LRPC: CAPRA 0-2<br>IRPC: CAPRA 3-5	NA	Every 1-2 year	NA

NCCN – National Comprehensive Cancer Network, CAPRA - Cancer of the Prostate Risk Assessment

Table S2. Baseline characteristics of the different study populations.

Study name	Risk group	Number of patients (percent)	Age (years)	PSA	GS			cT stage				PSA density
					3+3	3+4	4+3	cT1	cT2a	cT2b	cT2c	
Ventimiglia (2022 Sep, n=16177) [24]	VLRPC	5522 (34)	67 (62-71)	5.6 (4.1-7.8)	14 684 (91)	1118 (7)	0	13715 (85)	2370 (14.6)			NA
	LRPC	9501 (59)										
	IRPC	1154 (7)										
Cyll (2022 Apr, n=358) [25]	LRPC	177 (49)	63 (58-68)	NA	177 (100)	0	0	129 (73)	48 (27)			0.13 (0.09-0.19)
	IRPC	181 (51)	66 (61-70)		54 (30)	127 (70)	0	126 (70)	55 (30)			0.18 (0.13-0.26)
Courtney (2022 Feb, n=9733) [26]	LRPC	8726 (89)	65.1 (61.4-68.9)	5.35 (4.20-6.6)	8726 (100)	0	0	7544 (86)	1182 (14)			NA
	FIRPC	773 (8)	65.6 (61.6-69.8)	8.03 (4.80-11.0)	369 (48)	404 (52)	0	592 (77)	181 (23)			
	UIRPC	234 (3)	66.3 (61.1-71.3)	8.63 (5.05-11.7)	9 (4)	82 (35)	143 (61)	152 (65)	82 (35)			
Sayyid (2022 Jan, n=20334) [27]	FIRPC	20334 (100)	64.0 (58.0-69.0)	5.60 (4.40-7.70)	11110 (55)	9924 (45)	0	9603 (47)	2173 (11)	373 (2)	8185 (40)	NA
Cooley (2021 Nov, n=6775) [28]	LRPC-LV	4604 (68)	64.0 (58.0-68.2)	5.0 (3.7-6.7)	6207 (92)	482 (7)	81 (1)	5387 (80)	870 (13)			NA
	LRPC-HV	360 (5)										
	IRPC	1288 (19)										
Herden (2021 Jul, n=329) [29]	VLRPC	207 (63)	69.0 (63.4-72.5)	5.3 (3.9-7.2)	307 (93)	21 (6)	0	277 (84)	36 (11)	9 (3)	7 (2)	NA
	LRPC	70 (21)	68.2±7.5									
	IRPC*	52 (16)	69.3±6.8									
Rakauskas (2021 Jul, n=51) [30]	LRPC	17 (33)	64 (60-69)	5.2 (3.4-6)	17 (100)	0	0	16 (94)	1 (7)			0.11 (0.1-0.2)
	FIRPC	34 (67)	66 (62-69)	5.6 (4.5-8.1)	26 (76)	8 (24)	0	30 (88)	4 (12)			0.18 (0.1-0.3)

Mukherjee (2021 Jan, n=372) [31]	LRPC	276 (74)	63.6±6.6	5.7 (4.2–7.0)	276 (100)	0 (0)	0	229 (83)	47 (17)	0	0	0.11 (0.09, 0.14)
	IRPC	96 (26)	65.5±6.7	7.3 (4.2-10.8)	41 (43)	55 (57)	0	70 (73)	23 (24)	3 (3)	0	0.15 (0.10, 0.21)
Carlsson (2020 Jun, n=219) [32]	IRPC	219 (100)	67 (61-72)	5 (4-7)	0	219 (100)	0	151 (69)	31 (14)	5 (2)	3 (1)	NA
Richard (2020 Jun, n=374) [33]	IRPC	374 (100)	67±8	6.9 (5.2-9.5)		283 (76)	91 (24)	NA	NA	NA	NA	NA
Butler (2019 Sep, n=15603) [34]	LRPC	12380 (79)	NA	NA	NA	NA	NA	NA	NA	NA	NA	NA
	FIRPC	2005 (13)										
	UIRPC	1218 (8)										
Shelton (2019 Aug, n=548) [35]	VLRPC	218 (40)	NA	5.2 (4.3-6.8)	515 (94)	29 (5)	4 (1)	433 (79)	81 (15)	3 (1)		NA
	LRPC	259 (47)										
	IRPC	71 (13)										
Thomsen (2019 Mar, n=963) [36]	VLRPC	223 (24)	66 (63.1-68.1)	6.7 (5.2-9.2)	841 (90)	85 (9)	10 (1)	798 (83)	106 (11)	20 (2)	12 (1)	0.15 (0.10-0.21)
	LRPC	436 (47)										
	IRPC	259 (28)										
Masic (2018 Oct, n=1243) [37]	LRPC	1119 (90)	62±7.3	5.4 (4.2-7.3)	1119 (90)	124 (10)	0	873 (71)	361 (29)			0.13 (0.09-0.18)
	IRPC	124 (10)										
Whalen (2018 Apr, n=237) [38]	LRPC	175 (74)	69.8±8.4	5.6±3.6	155 (65)	75 (32)		205 (86)	33 (14)			NA
	IRPC	62 (26)										
Thostrup (2018 Feb, n=451) [15]	VLRPC	152 (34)	65.6 (63.0-68.0)	6.7 (5.2-9.1)	412 (91.4)	38 (8.4)	1 (0.2)	408 (91)	38 (8)	4 (0.9)	1 (0.1)	0.14 (0.09-0.21)
	LRPC	183 (41)										
	IRPC	111 (25)										
Savdie	VLRPC	245 (37)	62.9 (42-82)	5 (0.5-9.8)	245 (100)	0	0	228 (93)	0			0.11 (0.01 0.15)

(2017 Oct, n=651) [39]	LRPC	262 (40)	64.4 (39-79)	5.38 (0.33-10)	264 (100)	0	0	99 (38)	156 (59)			0.16 (0.01-0.87)
	IRPC	144 (22)	67.2 (45-83)	8.5 (0.3-46)	78 (55)	55 (38)	10 (7)	81(54)	58 (41)			0.18 (0.04-0.71)
Nyame (2017 Sep, n=631) [40]	LRPC*	514 (81)	65.1 (60.2-69.1)	4.8 (3.5-6.3)	514 (100)	0	0	NA	NA			0.11 (0.08-0.16)
	IRPC**	117 (18)	68.6 (63.7-73.8)	8 (5-11.8)	39 (33)	67(57)	11(9)					0.15 (0.11-0.27)
Musunuru (2016 Dec, n=945) [41]	LRPC	732 (77)	67 (60.6-71.9)	4.8 (3.2-6.6)	732 (100)	0	0	614 (84)	96 (13)			NA
	IRPC	213 (23)	72 (67.3-76.8)	10.1 (6.2-11.6)	85 (39)	102 (48)	20 (9)					
Godtman (2016 Nov, n=474) [14]	VLRPC	244 (51)	66.0 (63.1-68.1)	NA	NA	NA	NA	NA	NA			NA
	LRPC	126 (27)										
	IRPC	104 (22)										
Berg (2016 May, n=235) [42]	LRPC	178 (76)	66	4.1	178 (76)	29 (12)	6 (3)	NA	NA			NA
	IRPC	35 (15)										
Yamamoto (2016 May, n=980) [43]	LRPC	769 (78)	70	6.2	847 (86)	133 (14)		783 (80)	167 (17)			NA
	IRPC	211 (22)										
Loeb (2015 Feb, n=1729) [44]	VLRPC	644 (37)	64.0 (60.0-67.0)	5.6 (4.1-8.0)	514 (30)	116 (7)	0	1497 (87)	232 (13)			NA
	LRPC	757 (44)										
	IRPC	328 (19)										
Bul (2012 Dec, n=509) [11]	LRPC	381 (74)	67.6 (64.2-71.3)	4.1 (3.2-5.0)	381 (100)	0	0	325 (85)	48 (12)	3 (1)	5 (1)	0.10 (0.07-0.13)
	IRPC	128 (26)	67.4 (64.7-72.1)	5.3 (4.0-7.6)	99 (77)	25 (20)	4 (3)	104 (81)	19 (15)	5 (4)	0	0.20 (0.12-0.25)
Cooperberg (2011 Jan, n=466) [45]	LRPC	376 (81)	62	4.99	376 (100)	0	0	247 (66)	129 (34)			NA
	IRPC	90 (19)	65	10.30	61 (68)	27 (30)	2 (2)	56 (62)	34 (38)			

Summary statistics are as follows: mean ± standard deviation, median (interquartile range), or number (%).

*Also includes some high-risk patients.

** 213 (41%) patients were VLRPC.

*** 9 (8%) patients were HRPC.

VLRPC – Very low risk prostate cancer, LRPC – Low risk prostate cancer, IRPC – Intermediate risk prostate cancer, FIRPC – Favourable intermediate risk prostate cancer, UIRPC – Unfavourable intermediate risk prostate cancer, LRPC- LV – Low risk prostate cancer – low volume, LRPC- HV – Low risk prostate cancer – high volume.

Table S3. Follow up period and survival data of the different study populations.

Study name	Risk group	Number of patients (percent)	Follow up period in years	Definitive treatment	Treatment free survival	Metastasis free survival	Cancer specific survival	Overall survival
Ventimiglia (2022 Sep, n=16177) [24]	VLRPC	5522 (34)	NA	NA	NA	NA	NA	NA
	LRPC	9501 (59)						
	IRPC	1154 (7)						
Cyll (2022 Apr, n=358) [25]	LRPC	177 (49)	4.2 (2.3-6.0)	65 (40)	5yrs 69 (61-76)	NA	NA	5yrs 97 (95-98)
	IRPC	181 (51)		97 (60)	5yrs 44 (36-52)			
Courtney (2022 Feb, n=9733) [26]	LRPC	8726 (89)	7.6 (5.7-9.9)	3575 (40)	10yrs 55.1 (53.9-56.3)	10yrs 98.5 (98.1-98.8)	10yrs 98.9 (98.6-99.2)	10yrs 76.8 (75.6-78)
	FIRPC	773 (8)	7.6 (5.8-9.8)	607 (78)	10yrs 18.4 (15.7-21.7)	10yrs 90.4 (87.5-92.9)	10yrs 96.3 (94.3-97.7)	10yrs 73.8 (69.4-78)
	UIRPC	234 (3)	7.8 (6.2-9.5)	179 (76)	10yrs 21.5 (16.4-27.8)	10yrs 80.8 (74.1-86.7)	10yrs 88.2 (81.6-93.2)	10yrs 59.4 (50.7-68.3)
Sayyid (2022 Jan, n=20334) [27]	FIRPC	20334 (100)	NA	17 895 (88)	NA	NA	NA	NA
Cooley (2021 Nov, n=6775) [28]	LRPC- LV	4604 (68)	6.8	1195 (26)	5yrs 78.6 (77.4-79.9)	NA	NA	NA
	LRPC- HV	360 (5)	5.9	229 (64)	5yrs 35.8 (30.5-41.9)			
	IRPC	1288 (19)	6.1	493 (38)	5yrs 64.1 (61.3-67)			
Herden (2021 Jul, n=329) [29]	VLRPC	207 (63)	7.7 (4.7-9.1)	108 (52)	NA	NA	NA	NA
	LRPC	70 (21)		45 (64)				
	IRPC*	52 (16)		34 (65)				
Rakauskas	LRPC	17 (33)	3	0	NA	NA	NA	NA

(2021 Jul, n=51) [30]	FIRPC	34 (67)		17 (50)				
Mukherjee (2021 Jan, n=372) [31]	LRPC	276 (74)	4.9 (2.6-7.8)	86 (31.2)	5yrs 63 (55-69)<br>10yrs 54 (44-62)	NA	NA	5yrs 93 (88-96)<br>10yrs 90 (83-94)
	IRPC	96 (26)	4.1 (2.2-6.1)	22 (23)	5yrs 69 (56-79)<br>10yrs 69 (56-79)			5yrs 93 (81-97)<br>10yrs 80 (50-93)
Carlsson (2020 Jun, n=219) [32]	IRPC	219 (100)	3.1 (1.9-4.9)	64 (29)	5yrs 61 (52-70)<br>10yrs 49 (37-60)	NA	NA	5yrs 97 (93-99)<br>10yrs 77 (48-92)
Richard (2020 Jun, n=374) [33]	IRPC	374 (100)	8.1 (6.0-10.1)	266 (71)	1yr 69.5<br>5yrs 34.9	NA	5yrs 98<br>8yrs 94	5yrs 94<br>8yrs 82
Butler (2019 Sep, n=15603) [34]	LRPC	12380 (79)	NA	NA	NA	NA	5yrs 99.9	5yrs 96.3
	FIRPC	2005 (13)					5yrs 99.0	5yrs 93.0
	UIRPC	1218 (8)					5yrs 98.7	5yrs 87.2
Shelton (2019 Aug, n=548) [35]	VLRPC	218 (40)	3.4	50 (29)	NA	NA	NA	NA
	LRPC	259 (47)		92 (54)				
	IRPC	71 (13)		29 (17)				
Thomsen (2019 Mar, n=963) [36]	VLRPC	223 (24)	7.5 (6.1-9.1)	320 (34)	5yrs 73.4 (67.0-78.7)<br>10yrs 70.8 (64.0-76.5)	NA	NA	5yrs 95.5 (91.8-97.6)<br>10yrs 88.6 (81.5-93.2)
	LRPC	436 (47)			5yrs 64.5 (59.8-68.8)<br>10yrs 55.7 (49.9-61.0)		10yrs 99.3 (97.3-99.8)	5yrs 95.2 (92.7-96.8)<br>10yrs 87.9 (83.5-91.2)
	IRPC	259 (28)			5yrs 73.5 (67.6-78.5)<br>10yrs 69.0 (61.8-75.0)		10yrs 99.5 (96.6-99.9)	5yrs 95.8 (92.5-97.6)<br>10yrs 83.9 (75.1-89.7)
Masic	LRPC	1119 (90)	5.2	NA	5yrs 64	5yrs 99	NA	NA

(2018 Oct, n=1243) [37]	IRPC	124 (10)			5yrs 49	5yrs 98		
Whalen (2018 Apr, n=237) [38]	LRPC	175 (74)	6.0	68 (39)	NA	NA	NA	NA
	IRPC	62 (26)		27 (44)				
Thostrup (2018 Feb, n=451) [15]	VLRPC	152 (34)	5.1 (4.6-5.6)	142 (32)	5yrs 62.1 (51.9-72.3)	NA	NA	NA
	LRPC	183 (41)			5yrs 54.0 (45.4-62.6)			
	IRPC	111 (25)			5yrs 70.9 (61.1-80.7)			
Savdie (2017 Oct, n=651) [39]	VLRPC	245 (37)	4.5 (2.8-7.1)	79 (32.2)	5yrs 66.6<br>10yrs 45.5	NA	5yrs 100<br>10yrs 100	5yrs 98.6 (81-97)<br>10yrs 94.1 (50-93)
	LRPC	262 (40)	4.5 (2.4-7)	115 (43.8)	5yrs 55.5<br>10yrs 38.8			
	IRPC	144 (22)	4.4 (2.6-6.7)	65 (41)	5yrs 50.0<br>10yrs 34.1			
Nyame (2017 Sep, n=631) [40]	LRPC**	514 (81)	4.2 (2.5-6.7)	182 (35)	5yrs 62.0 (57-66.6)<br>10yrs 48.7 (41.1-55.8)	5yrs 99.2 (96.6-99.8)<br>10yrs 97.4 (89.6-99.4)	100%	5yrs 98.4 (96.2-99.3)<br>10yrs 96.5 (92.9-98.3)
	IRPC***	117 (18)	3.6 (2.6-6)	40 (34)	5yrs 58.8 (46.4-68.7)<br>10yrs 52.3 (38.9-64.1)	5yrs 99.0 (93.2-99.9)<br>10yrs 99.0 (93.2-99.9)	100%	5yrs 95.6 (86.9-98.6)<br>10yrs 77 (34.4-93.8)
Musunuru (2016 Dec, n=945) [41]	LRPC	732 (77)	6.5 (3.8-9.1)	547 (74)	10yrs 64 (60-69)<br>15yrs 58 (52-65)	10yrs 96 (94-98)<br>15yrs 95 (91-98)	10yrs 98 (97-100)<br>15yrs 97 (93-100)	10yrs 84 (80-88)<br>15yrs 67 (59-75)
	IRPC	213 (23)	6.7 (3.9-10.4)	139 (65)	10yrs 61 (54-69)<br>15yrs 48 (38-61)	10yrs 91 (85-97)<br>15yrs 82 (73-92)	10yrs 97 (94-100)<br>15yrs 89 (80-98)	10yrs 67 (60-76)<br>15yrs 51 (41-63)
Godtman (2016 Nov, n=474) [14]	VLRPC	244 (51)	6.3	202 (43)	10yrs 53 (44-61)<br>15yrs 48 (39-57)	10yrs 99 (96-100)<br>15yrs 93 (84-97)	10yrs 100<br>15yrs 100	10yrs 80 (76-84)<br>15yrs 51 (42-59)
	LRPC	126 (27)			10yrs 42 (31-52)<br>15yrs 27 (13-43)		10yrs 100<br>15yrs 94 (77-98)	

	IRPC	104 (22)			10yrs 41 (28-53)<br>15yrs 13 (1-37)		10yrs 98 (85-100)<br>15yrs 90 (72-97)	
Berg (2016 May, n=235) [42]	LRPC	178 (76)	3.5	18 (10)	2yrs 94<br>5yrs 82<br>10yrs 67	NA	NA	NA
	IRPC	35 (15)		7 (20)				
Yamamoto (2016 May, n=980) [43]	LRPC	769 (78)	6.4	NA	NA	5yrs 100<br>10yrs 95<br>15yrs 92	NA	NA
	IRPC	211 (22)				5yrs 96<br>10yrs 90<br>15yrs 84		
Loeb (2015 Feb, n=1729) [44]	VLRPC	644 (37)	5	614 (36)	1yr 97<br>2yrs 85<br>3yrs 77<br>4yrs 70<br>5yrs 65	NA	NA	NA
	LRPC	757 (44)			1yr 96<br>2yrs 87<br>3yrs 79<br>4yrs 73<br>5yrs 67			
	IRPC	328 (19)			1yr 94<br>2yrs 82<br>3yrs 71<br>4yrs 63<br>5yrs 59			
Bul (2012 Dec, n=509) [11]	LRPC	381 (74)	7.5 (4.9-9.6)	152 (39)	10yrs 49.7	10yrs 99.7	10yrs 99.1	10yrs 79
	IRPC	128 (26)	7.2 (5.3-9.9)	69 (54)	10yrs 30.3	10yrs 96.4	10yrs 96.1	10yrs 64.5
Cooperberg (2011 Jan, n=466) [45]	LRPC	376 (81)	3.9	113 (30)	NA	NA	NA	NA
	IRPC	90 (19)	4.3	31 (35)				

Summary statistics are as follows: number (%), median (interquartile range), or % (95% confidence interval).

*Also includes some high-risk patients.

** 213 (41%) patients were VLRPC.

*** 9 (8%) patients were high-risk.

VLRPC – Very low risk prostate cancer, LRPC – Low risk prostate cancer, IRPC – Intermediate risk prostate cancer, FIRPC – Favourable intermediate risk prostate cancer, UIRPC – Unfavourable intermediate risk prostate cancer, LRPC- LV – Low risk prostate cancer – low volume, LRPC- HV – Low risk prostate cancer – high volume.
